# Getting specific: participation preference in urooncological decision-making

**DOI:** 10.1186/s12911-023-02201-8

**Published:** 2023-07-06

**Authors:** Björn Büdenbender, Anja K. Köther, Maximilian C. Kriegmair, Britta Grüne, Maurice S. Michel, Georg W. Alpers

**Affiliations:** 1 Department of Psychology, School of Social Sciences, University of Mannheim, L 15-17, 68131 Mannheim, Germany; 2 Department of Urology and Urosurgery, University Medical Center Mannheim, University of Heidelberg, Mannheim, Germany

**Keywords:** Shared decision-making, Decision context, Patient participation, Patient preference, Participation preference, Oncology, Urology, Bladder cancer

## Abstract

**Background:**

Shared decision-making is the gold standard for good clinical practice, and thus, psychometric instruments have been established to assess patients’ generic preference for participation (e.g., the Autonomy Preference Index, API). However, patients’ preferences may vary depending on the specific disease and with respect to the specific decision context. With a modified preference index (API-Uro), we assessed patients’ specific participation preference in preference-sensitive decisions pertaining to urological cancer treatments and compared this with their generic participation preference.

**Methods:**

In *Study 1,* we recruited (*N* = 469) urological outpatients (43.1% urooncological) at a large university hospital. Participation preference was assessed with generic measures (API and API case vignettes) and with the disease-specific API-Uro (urooncological case vignettes describing medical decisions of variable difficulty). A polychoric exploratory factor analysis was used to establish factorial validity and reduce items.

In *Study* 2, we collected data from *N* = 204 bladder cancer patients in a multicenter study to validate the factorial structure with confirmatory factor analysis.

Differences between the participation preference for different decision contexts were analyzed.

**Results:**

*Study 1*: Scores on the specific urooncological case vignettes (API-Uro) correlated with the generic measure (*r* = .44) but also provided incremental information. Among the disease-specific vignettes of the API-Uro, there were two factors with good internal consistency (α ≥ .8): treatment versus diagnostic decisions. Patients desired more participation for treatment decisions (77.8%) than for diagnostic decisions (22%), χ^2^(1) = 245.1, *p* ≤ .001.

*Study 2:* Replicated the correlation of the API-Uro with the API (*r* = .39) and its factorial structure (SRMR = .08; CFI = .974). Bladder cancer patients also desired more participation for treatment decisions (57.4%) than for diagnostic decisions (13.3%), χ²(1) =84, *p* ≤ .001.

**Conclusions:**

The desire to participate varies between treatment versus diagnostic decisions among urological patients. This underscores the importance of assessing participation preference for specific contexts. Overall, the new API-Uro has good psychometric properties and is well suited to assess patients’ preferences. In routine care, measures of participation preference for specific decision contexts may provide incremental, allowing clinicians to better address their patients’ individual needs.

**Supplementary Information:**

The online version contains supplementary material available at 10.1186/s12911-023-02201-8.

## Background

Shared decision-making (SDM) is now considered the gold standard for good patient-physician interaction [1] and a stipulated goal for quality improvement in most modern healthcare systems [2, 3]. SDM emphasizes the importance of the patient’s voice and embraces their right to self-determination, and is thus an ethical imperative [4]. Over the last two decades, evidence has emerged that patient participation in medical decision-making can yield positive effects [5], such as lower emotional distress for the patient [6, 7], higher satisfaction with the decisions, better interaction with the physician, and improved knowledge [8–10].

SDM is especially relevant for preference-sensitive decisions [11], where the most appropriate decision can only be determined by taking the patient’s preferences into account [12]. Such preference-sensitive decisions need to be made when there is scientific uncertainty, i.e., different treatment options are from a professional perspective in equipoise (equally effective) or when the decision substantially affects patients’ subsequent quality of life [13–15].

Patients in urooncological care are regularly confronted with demanding and ambiguous, thus preference-sensitive decisions. For example, treatment of early-stage prostate cancer requires patients and physicians to jointly deliberate multiple treatment options in equipoise: active surveillance, surgery, and several forms of radiation therapy [11, 16]. Another prime example of preference-sensitive decisions in urooncology is the choice of urinary diversion after cystectomy (the surgical removal of the bladder) [17], where only a small fraction of patients receives a clear cut-recommendation for a specific type of urinary diversion based on their medical parameters [18, 19]. Given the inherent preference-sensitive nature of urooncological decisions and the promising evidence for the positive effects of patient participation in these decisions, international urooncological treatment guidelines now explicitly recommend investing in patient participation [20, 21].

On the other hand, not all patients seek autonomy in medical decision-making to the same degree [22–24]; some authors even described a desire for dependency in some of their patients [25]. Either way, patients’ preference for autonomy varies substantially, depending on the context of a decision. For example, participation preference wanes with more severe diagnoses in case vignettes of varying illness severity [23]. A recent study found considerable differences between the desire for autonomy in generic medical decision-making and disease-specific decisions [26]. Thus, there is a need for psychometrically sound measures to assess patients’ desire for autonomy in the specific decision-making context of the treatment decisions they face, e.g., for the highly preference-sensitive decisions in the context of urological cancer treatment.

Previous research has identified at least two relevant dimensions of patients’ desire for autonomy: A) the preference for information-seeking and B) the preference for participation in decision-making [23]. While many studies have documented that patients unanimously have a high preference for information-seeking [27, 28], there are considerable inter and intra-individual differences in the preference for active participation in the decision-making process (i.e., participation preference).

Patients’ participation preferences have previously been assessed with several measures in various samples from many cultures, and findings have been systematically aggregated in seven reviews from 1998 to 2021 [27, 29–34]. Concerning urooncology, these reviews included nine studies on prostate cancer populations, with the amount of patients’ desiring active participation ranging from 42% [35] to 92.5% [36], and four studies on the decision context of prostate cancer screening, with the proportion of preference for active participation ranging from 64.4% [37] to 81% [38].

Given the previously outlined high variability in participation preference for different decision contexts and diagnoses, it appears imperative to assess participation preference in the specific decision context. Therefore, in light of the highly preference-sensitive decisions urooncological patients face, we set out to develop a measure to determine preference for participation in the specific context of the most important urooncological treatment decisions.

Currently, patients’ preferences for participation in decision-making are not routinely collected at the outset of urooncological treatments. However, such an assessment could inform individually tailored healthcare and pave the way for high-quality decisions [39]. Practitioners could be enabled to involve their patients in the decision-making process according to their true preferences rather than relying on assumptions derived from patients’ sociodemographic characteristics [40].

The consistent finding that physicians across disciplines appear to have difficulties to accurately predict their patients’ participation preferences highlights the benefit of a formal assessment with disease and context-specific questionnaires [41–43]. An accurate assessment of participation preference is a prerequisite for a proper SDM process. When patients feel obliged to exercise more autonomy than they prefer, it could induce or exacerbate negative feelings, such as being overwhelmed, afraid, or abandoned, and lead to significant decision regret [25, 33]. Assessing patients’ participation preferences for the specific decision context and prior to the decision situation could aid physicians in navigating how much involvement their patients want and prevent overwhelming the patients [44].

The primary endpoint of this research was to capture disease-specific participation preference and determine its potential incremental value. To this end, we developed an assessment instrument to measure specific preferences in the context of urooncological decision-making. After confirming its incremental value and good psychometric properties, it will be available for clinical practice. In order to increase the usefulness of the instrument for clinical practice, we took great care to devise a short and easy to comprehend scale. Moreover, we collected data from two populations in two independent studies in order to examine generalizability respectively usefulness in a broad range of clinical settings.

Although there are some studies on the autonomy preference in prostate cancer patients, as summarized above, there are, to the best of our knowledge, no studies about the autonomy preference in other common urooncological entities (e.g., bladder cancer). This lack of research is striking, given the imperative to implement SDM in urooncology due to the many preference-sensitive decisions these patients face. Thus, a secondary endpoint of our study was to contribute to the knowledge base on participation preference in previously neglected populations.

## Methods

### Samples and procedures

#### Urological cohort (Study 1)

Data collection was carried out between May 2019 and February 2021 as part of an SDM implementation study in general urological practice. We collected data at the outpatient clinic of the Department of Urology and Urosurgery at University Medical Center Mannheim, Germany. Eligible patients were at least 18 years old and fluent in German. While waiting for their consultation with the physician, they were informed about the study by a nurse. After providing informed consent, they were then asked to fill in a set of questionnaires.

Patients filled in the German Autonomy Preference Index (API; [45]), the API’s original vignettes (API-OV; [23]), and the newly developed urooncological case vignettes (API-Uro; [46]; see Sect. "Development of the urooncological case vignettes (API-Uro)" and Additional file 1: Appendix A). Further questionnaires on emotional distress, decisional conflict, patients’ attitudes and beliefs, and perceived participation were assessed and analyzed elsewhere (see [47–49] for more details). The study protocol was approved by the ethics committee of the Medical Faculty of Mannheim, University of Heidelberg (MA-2019-635N).

We recruited *N* = 502 urological patients (age > 18). We excluded 33 patients (6.6%) with more than 40% missing values. The remaining 469 patients were primarily male (86.8%), of older age (62.4 ± 13.6 years), and most were German nationals (97.2%). Due to the absence of any systematic pattern of missing values (Additional file 1: Figures B.1 and B.2), we used median imputation for cases where only one item was missing per questionnaire. We imputed 24 values (5.1%) for the API-OV and 26 values (5.5%) in the API-Uro questionnaire.

#### Bladder cancer cohort (Study 2)

In addition to the methods described above, we obtained data in a multicenter study with bladder cancer patients scheduled for radical cystectomy before their pre-treatment consultation. Data were collected between September 2019 and February 2022 in the urology departments of six independent German hospitals. Inclusion criteria and procedure were identical to the *urological cohort study* (*Study 1)* and equivalent in all six study centers. A study nurse approached patients, and after giving informed consent, they completed a set of self-report measures, including the German API [45], the original case vignettes API-OV [23], and the urooncological case vignettes API-Uro [46]. More details on the study procedure and additional measured constructs can be found in Köther et al. [50]. The study was approved by the ethics committee of the Medical Faculty of Mannheim of the University of Heidelberg (MA-2019-727N).

A total of *N* = 223 bladder cancer patients participated in the study. We excluded *n* = 19 patients with more than 40% missing data. Patients in the final sample (*N* = 203) were, on average, 68.4 ± 9 years old, and the majority were male (74.5%) and German nationals (87.3%). Again, visual inspection of the response pattern for the generic, original vignettes API-OV (see Additional file 1: Figure B.3) and the urooncological case vignettes (API-Uro, see Additional file 1: Figure B.4) did not show any systematic pattern of missing values. Thus, median imputation was applied for cases with only one missing value. We imputed 13 (5.9%) values in the API-OV and 10 (4.9%) in the API-Uro.

### Measures

The primary endpoint of our study was to assess and evaluate the relevance of specific measures of participation preference. Concomitantly, we aimed for a rigorous psychometric evaluation of the urooncology-specific participation preference measure, which we assessed in two independent study populations (Study 1 and Study 2). Although populations and procedures differed, the same set of measures was employed in both studies to increase comparability. They are, thus, outlined once for both studies.

#### Sociodemographic and clinical data

We collected information on the following sociodemographic patient characteristics: age, gender, highest educational level, occupational status, relationship status, and cohabitation. Furthermore, we retrieved patients’ primary diagnoses from their electronic health records. For the *urological cohort (Study 1),* primary diagnoses were dichotomized into non-oncological vs. oncological. We further categorized oncological diagnoses according to the cancer entities (prostate cancer, renal cancer, bladder cancer, and others).

#### Preference for participation in decision-making

##### Autonomy Preference Index (API)

The API [23] is an established, well-validated questionnaire that has been used in multiple contexts to assess the generic participation preference of patients [51]. The German version of the API consists of eleven Likert-response type items, with a five-point scale ranging from 0 “*strongly disagree*” to 4 “*strongly agree*” [45]. The API has two subscales: the information-seeking subscale (API-is; seven items) and the decision-making preference scale (API-dm; four inversed items). Sum scores for both scales are built, and for better interpretation, min–max normalized to range from 0 to 100, with higher values indicating a stronger desire for autonomy.

##### Original case vignettes of the API (API-OV)

In the original English version of the API [23], the decision-making subscale is supplemented with three vignettes (upper respiratory tract illness, high blood pressure, myocardial infarction). One of the authors (BB) translated the vignettes, and an independent bilingual speaker not associated with the project back-translated them. Deviations from the back-translated version to the original were revised until a consensus was reached.

Each vignette contains three items (putative decisions). Patients were asked to indicate who should make the corresponding decision for each of the nine items. Options were presented with a five-point Likert response format from 1 “*physician alone*” to 5 “*patient alone*”. We calculated a sum score with all nine items and transformed it with min–max normalization to range from 0 to 100. Higher values are indicative of a preference for more decision-making autonomy.

##### Development of the urooncological case vignettes (API-Uro)

Based on the API-OV vignettes, we constructed a measure for specific urooncological participation preference. First, an expert panel created an initial pool of 18 items (putative decisions). Items (putative decisions) were formulated based on German cancer aid’s patient guidelines for urooncological cancer entities, available at the foundation’s website [52]. The panel then clustered the items to represent six essential steps from initial diagnosis, through critical treatment decisions, to aftercare. Next, we conducted multiple rounds of revisions and incorporated the feedback of four patients who participated in a pilot test. As a result, item complexity was reduced, the instructions were simplified, and redundant or ambiguous items were removed. The resulting preliminary questionnaire contained six vignettes corresponding to twelve items.

The response format remained identical to the API-OV. An English translation of the final version of the questionnaire used in this study is available in Additional file 1: Appendix A. We built a total sum score for the API-Uro and transformed it with min–max normalization to range from 0 to 100. A higher score indicates a greater desire to participate. In addition, we calculated sum scores for the two factors we obtained during the psychometric evaluation of the questionnaire described below.

### Statistical analyses

We conducted drop-out analyses in both studies using either Pearson χ^2^ test, Fisher’s exact test, independent sample Student’s *t*-test, or, in case of homogeneity of variances, Welch’s two-sample *t*-test. The psychometric evaluation of the API-Uro questionnaire involved the following consecutive steps: determining the factorial validity with a polychoric exploratory factor analysis (EFA) (*urological cohort; Study 1*), validation of the established factor structure with confirmatory factor analysis (CFA) in the *bladder cancer cohort (Study 2)*, assessment of reliability in terms of internal consistency (ordinal coefficient α), and evaluation of construct validity of the API-Uro (both studies).

All psychometric analyses described above building upon a correlation matrix between the Likert response-format items (parallel analysis, EFA and CFA, Cronbach’s α) were calculated with polychoric correlations. We followed best practice recommendations [53–55] and made the following informed choices: EFA using minimal residual (minres) extraction method, with an oblique rotation (oblimin). As there is no single best technique to determine the optimal number of factors to retain, we compared results from multiple methods [54, 55]. To this end, we report results for the scree test, parallel analysis, VSS criterion, and Velicer’s MAP test.

The adequacy of the data was determined by inspection of the polychoric correlation matrix. The majority of inter-item correlations should fall between 0.3 and 0.7 [56]. We further checked the eligibility of our data for EFA with the Kaiser–Meyer–Olkin measure of sample adequacy (MSA; Cut-Offs 0.6 = mediocre, 0.7 = middling, 0.8 = meritorious, 0.9 = marvelous [57]) based on the polychoric correlation matrix as well as Bartlett’s test of sphericity.

We tested the factorial structure obtained in the first study (*urological cohort; Study 1*) with ordinal confirmatory factor analysis (CFA) in our data from the second study (*bladder cancer cohort; Study 2)*. Parameters were estimated with the diagonally weighted least square (DWLS) estimator, which has been shown to be robust and can handle non-multivariate normal data [58–60]. The model is evaluated in terms of the following fit statistics: *RMSEA*, *SRMR, CFI*, and *TLI*. Reliability was reported in terms of ordinal coefficient α as an appropriate measure for internal consistency in ordinal data [61].

In order to analyze the difference between the desire to participate in different decision-making contexts obtained during factor analysis (API-Uro factors), we categorized patients as participators and delegators based on an established convention from the literature [30, 50, 62]: a score of ≤ 40 indicates the desire to delegate the decision (delegators), while a score > 40 indicates a preference for autonomy and participation in decision-making (participators). Differences in the proportion of participators between the two decision contexts (API-Uro factors) were analyzed with the McNemar χ^2^ test.

Where applicable, we calculated and reported effect sizes; the interpretation of these effect sizes refers to the taxonomy of Cohen [63]. All analyses were carried out in R version 4.1.1 [64], and data were preprocessed with the tidyverse R-packages [65]. We used the R-packages psych and datscience for the polychoric EFA [66, 67]. The CFA was conducted with the lavaan, and lavaanExtra R-packages [68, 69]. A complete list of all R-packages can be obtained in Additional file 1: Appendix C.

## Results

### Sample characteristics

Most patients in both samples were male, German nationals, retired, lived with their partners, and had children. The average age of patients in the *urological cohort (Study 1)* was 62.4 ± 13.6 years, and in the *bladder cancer cohort (Study 2),* 68.4 ± 9 years. Further details on both samples’ characteristics can be found in Table 1. The *urological cohort (Study 1)* contained 43.1% patients with an urooncological diagnosis (65.3% prostate cancer). All patients were diagnosed with bladder cancer in the *bladder cancer cohort (Study 2)*.

**Table 1:** Sociodemographic characteristics

Characteristic	*Urological Cohort (Study 1)* (*N* = 469)		*Bladder Cancer Cohort (Study 2)* (*N* = 204)	
	*n*	%	*n*	%
Sex^a^				
Male	407	86.8	152	74.5
Female	61	13.0	51	25.0
Highest educational level				
Without school graduation	3	0.6	5	2.5
Secondary school	192	40.9	137	67.2
High school	104	22.2	17	8.3
University	169	36.0	41	20.1
Employment status				
Unemployed	11	2.3	8	3.9
Student / Trainee	25	5.3	4	2.0
Employed	186	39.7	52	25.5
Retired	243	51.8	137	67.2
German national				
Yes	456	97.2	178	87.3
No	13	2.8	26	12.7
Cohabitation / marital status				
Living together with spouse/partner	367	78.3	155	76.0
Living separated from spouse/partner	14	3.0	2	1.0
Unmarried	40	8.5	7	3.4
Divorced	29	6.2	23	11.3
Widowed	19	4.1	15	7.4
Children				
Yes	355	75.7	173	84.8
No	112	23.9	30	14.7

Diverging cell counts from the total *N* are due to missing values still present after the median imputation

^a^The option “divers” was available but chosen by no patient

#### Drop-out analyses

We analyzed differences in sociodemographic variables between the final sample and patients who dropped out or had more than 40% missing values separately for both studies.

In the *urological cohort (Study 1),* the excluded patient (*n* = 33) contained significantly more non-German nationals (33.3% vs. 2.8%, χ^2^(1) = 63.1, *p* ≤ 0.001) with a moderate effect size of φ = 0.35, and more female patients (28% vs. 13%, χ^2^(1) = 63.1, *p* ≤ 0.035), with a small effect φ = 0.1. Moreover, we found differences in their educational level (*p* = 0.004, Fisher’s exact test). Excluded patients reported lower educational levels with a small effect size, Cramer’s *V* = 0.17. No significant differences existed in any other sociodemographic variable (all *p*s ≥ 0.08).

In the *bladder cancer cohort (Study 2),* there were no systematic differences between excluded patients (*n* = 15) and patients in the final sample (all *p*s ≥ 0.07).

#### Descriptive statistics of participation preference

Table 2 summarizes the descriptive statistics for the assessed participation preference measures. In both studies, patients scored very high on the information-seeking subscale of the API (API-is, *M*_study1_ = 95.8 ± 8.2, *M*_study2_ = 92.5 ± 11.6), and there was a ceiling effect. Patients scored in the moderate range for the measures of participation preference in decision-making (see Table 2). The total API-Uro scores were comparable to those obtained with the established generic API version and the original case Vignettes (API-OV). However, there were considerable differences between the factors (decision contexts), which can be differentiated with the API-Uro (see Sect. "Context specificity and urooncological participation preference").

**Table 2:** Descriptive Statistics for Participation Preference

Measure	*Urological Cohort (Study 1)* (*N* = 469)		*Bladder Cancer Cohort (Study 2)* (*N* = 204)	
	*n*	*Mean (SD)*	*n*	*Mean (SD)*
API-is^a^	465	95.8 (8.2)	170	92.5 (11.6)
API-dm^b^	459	44.6 (26.5)	167	30.5 (23.5)
API-OV^c^	442	34.6 (13.2)	161	30.0 (13.4)
API-Uro^d^	450	35.0 (13.4)	167	29.0 (13.7)

Diverging cell counts from the total *N* are due to missing values after the median imputation

^a^The information-seeking (*is*) subscale of the generic Autonomy Preference Index

^b^The decision-making (*dm*) subscale of the generic API

^c^Generic case vignettes from the original English API

^d^Total sum score of the final urooncological case vignettes, with seven items (decisions) on four vignettes

For the heterogeneous sample collected in the *urological cohort (Study 1),* we found no difference between urooncological and non-oncological patients in any of the participation preference measures (independent sample *t*-tests, all *p*s ≥ 0.141).

### Psychometric evaluation of the API-Uro

#### Factorial validity and reliability

We utilized the large dataset collected in the *urological cohort (Study 1)* to conduct a polychoric exploratory factor analysis (EFA) to assess the construct validity and reduce the number of ambiguous items in the API-Uro questionnaire. In preparation for the EFA, we first assessed the adequacy of our data. The polychoric correlations of the API-Uro items ranged between *r* = 0.23 and *r* = 0.89 (*Md* = 0.49); see Table 3. The majority of correlations for each item fell in the range between 0.3 ≤ *r* ≤ 0.7, indicating acceptable relatedness. Thus, no item was removed [56]. The data suitability was also confirmed by a meritorious KMO measure of sampling adequacy of 0.84 (Kaiser & Rice, 1974) and a significant Bartlett’s test, χ^2^(66) = 2871.8, *p* ≤ 0.001. All items had a KMO value of greater than 0.7 (range = [0.81; 0.90]); thus, no item was removed.

**Table 3:** Correlation Matrix and Descriptive Statistics for the API-Uro Items

Item	UV1.1	UV1.2	UV2.1	UV2.2	UV2.3	UV3.1	UV4.1	UV4.2	UV5.1	UV5.2	UV6.1	UV6.2
UV1.1	1.00											
UV1.2	0.88	1.00										
UV2.1	0.56	0.60	1.00									
UV2.2	0.44	0.53	0.75	1.00								
UV2.3	0.36	0.37	0.58	0.62	1.00							
UV3.1	0.29	0.29	0.44	0.39	0.45	1.00						
UV4.1	0.29	0.36	0.51	0.55	0.50	0.62	1.00					
UV4.2	0.30	0.36	0.46	0.54	0.35	0.38	0.69	1.00				
UV5.1	0.57	0.62	0.53	0.45	0.25	0.31	0.38	0.48	1.00			
UV5.2	0.54	0.60	0.48	0.44	0.22	0.33	0.37	0.44	0.89	1.00		
UV6.1	0.48	0.52	0.59	0.57	0.42	0.46	0.53	0.49	0.55	0.53	1.00	
UV6.2	0.45	0.49	0.49	0.51	0.35	0.43	0.50	0.52	0.55	0.52	0.85	1.00
Univariate Descriptive Statistics												
Median	2	2	3	3	3	3	3	3	2	2	3	3
Skew	-0.09	0.09	-0.49	-0.47	-0.13	-0.23	0.13	-0.11	0.22	0.13	-0.33	-0.42
Kurtosis	-0.47	-0.75	0.87	1.28	2.66	2.15	2.43	1.48	-0.80	-0.88	0.47	0.34
Missing^a^	8	9	2	0	0	1	4	9	4	5	9	8

*N* = 469. UV*i*.*k* = Urological Vignette, *i* indicates the vignette, *k* the item on the respective vignette (e.g., UV6.1, 6^th^ vignette 1^st^ item). All polychoric correlations are significant below *p* ≤ .001

^a^Missing values per item still present after the median imputation

The optimal number of factors to retain was determined by parallel analysis based on the polychoric correlations (five factors), scree test (two factors), VSS criterion (two factors), and Velicer’s Map test (one factor). The suggested five factors in the parallel analysis likely resemble over-factoring, which is a known problem for larger sample sizes [70] as the randomly generated Eigenvalues get too small. We decided to retain two factors in concordance with the unambiguous scree test (see Additional file 1: Figure D.1) and the VSS.

**Fig. 1:**
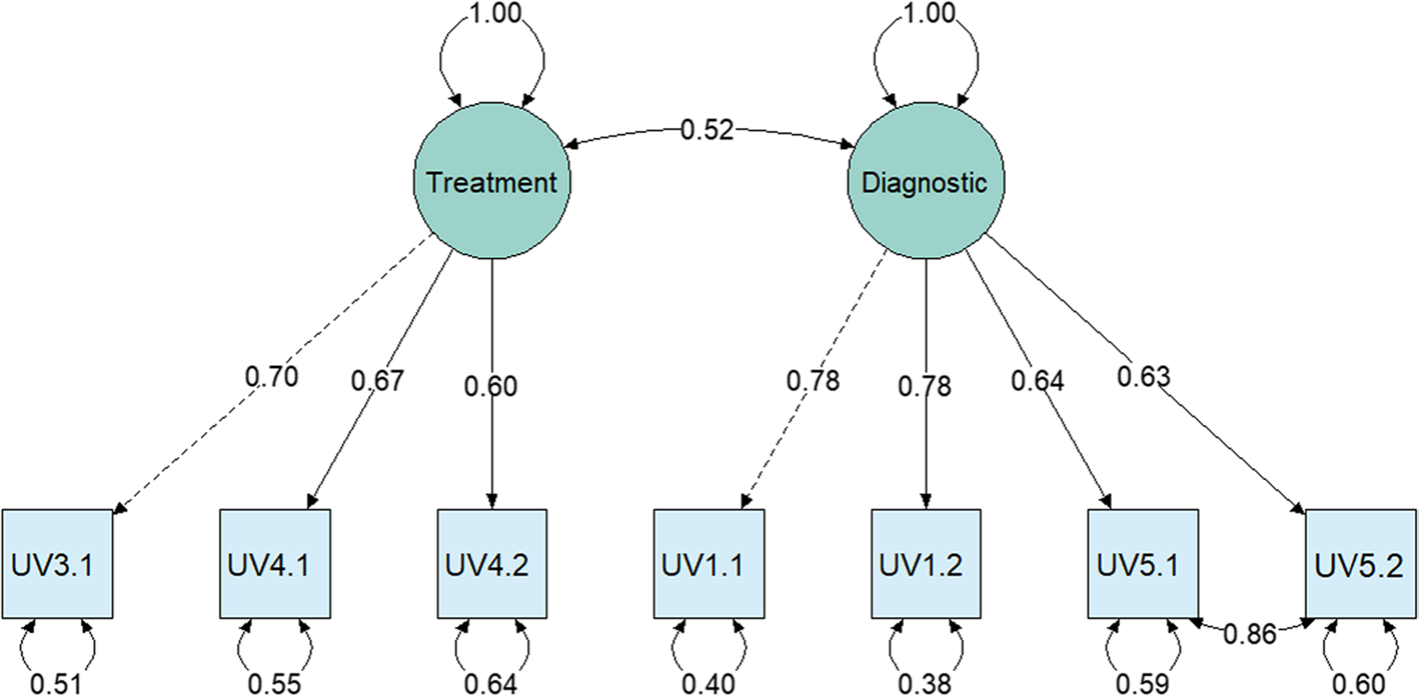
Confirmatory factor analysis of the API-Uro (Model 2) in the bladder cancer cohort (Study 2). *Note*. Loadings of the two latent factors (circles) on the seven manifest API-Uro items (squares). All coefficients are standardized. Loadings were extracted with a diagonally weighted least square estimator. UVi.k = Urological Vignette, i indicates the vignette, k the item on the respective vignette (e.g., UV5.1, 5^th^ vignette 1.^st^ item). After inspection of modification indices, the covariance between the residuals of items UV5.1 and UV5.2 was permitted in Model 2

Extraction of two factors with polychoric EFA with minimum residual (minres) extraction method and oblimin rotation explained 58.95% of the variance in the original twelve API-Uro items. The loadings of the items on the two factors are presented in Table 4. Two vignettes (UV2: “Form of therapy “ and UV6: “Palliative systemic therapy”) and their five corresponding items (UV2.1 – UV2.3, UV6.1, UV6.2) were eliminated due to substantial cross-loadings (≥ 0.3) of at least one item per vignette and in consolidation with the expert panel in order to shorten the questionnaire and increase its efficiency for practice.

**Table 4:** Loadings of API-Uro Items in Polychoric EFA

Item		F1: Treatment	F2: Diagnostic	*h*^*2*^	*u*^*2*^	*com*
UV1.1	*whether other procedures, such as a blood test or an ultrasound examination, are also applied?*		0.844	0.65	0.35	1.01
UV1.2	*whether there is an additional check-up with a urologist?*		0.879	0.75	0.25	1.00
UV2.1^a^	*whether a therapy takes place or the cancer is observed?*	**0.496**	**0.373**	0.60	0.40	1.86
UV2.2^a^	*whether surgery or radiotherapy is being performed?*	0.615		0.59	0.41	1.26
UV2.3^a^	*whether a new, experimental procedure is being used as part of a clinical trial?*	0.634		0.40	0.60	1.00
UV3.1	*whether an “radical” or “function-sparing” operation is carried out?*	0.681		0.42	0.58	1.02
UV4.1	*whether chemotherapy is administered?*	0.912		0.71	0.29	1.04
UV4.2	*when to start chemotherapy?*	0.665		0.49	0.51	1.02
UV5.1	*whether follow-up checks are necessary?*		0.77	0.68	0.32	1.03
UV5.2	*if so, how often should these take place?*		0.755	0.63	0.37	1.02
UV6.1^a^	*whether in case of side effects the therapy should be changed?*	**0.535**	**0.337**	0.61	0.39	1.69
UV6.2^a^	*whether in case of non-response the therapy should be changed?*	**0.491**	**0.332**	0.54	0.46	1.76

Exploratory factor analysis with minimum residual factor extraction and oblimin rotation. *N* = 465. Loadings ≤ .3 are omitted. Substantial cross-loadings (≥ .3, on two factors) are marked by boldface. *h*^*2*^ = communality; *u*^*2*^ = uniqueness; *com* = complexity

^a^Vignettes (UV2 and UV6) were excluded

The two vignettes “(Neo)adjuvant chemotherapy” (UV3) and “Functional preservation versus oncological safety” (UV4), loaded solely on factor one, while the remaining two vignettes, “Preventive care” (UV1) and “Follow-up care” (UV5) loaded exclusively on factor two. In consideration of the content of the respective vignettes, the factors were consequently termed *treatment decisions* (factor one, with vignettes UV3 and UV4) and *diagnostic decisions* (factor two, with vignettes UV1 and UV5). Standardized ordinal coefficients α were acceptable for the factor *treatment decisions* (α = 0.79) and good for *diagnostic decisions* (α = 0.9), as well as for the total sum scale (α = 0.92).

The psychometric analysis described above resulted in a shortened and final version of the API-Uro comprised of seven decisions presented in four vignettes: preventive care, functional preservation vs. oncological safety, (neo-) adjuvant chemotherapy, and follow-up care (see Additional file 1: Appendix A for the English version).

#### Validation of the factorial structure of the API-Uro

In order to validate the established two-factor structure of the API-Uro, we calculated a confirmatory factor analysis (CFA) with the diagonally weighted least square estimator in the multicenter sample of 204 patients from the *bladder cancer cohort (Study 2)*. Our first model (Model 1) was constructed to represent the factorial structure obtained with the polychoric EFA. In Model 1, the items UV3.1, UV4.1, and UV4.2 were expected to load on the *treatment decisions* (factor one) and the items UV1.1, UV1.2, UV5.1, and UV5.2 to load on the *diagnostic decisions* (factor two), with no covariance between the residuals. The standardized factor loadings for Model 1 ranged between 0.63 (UV1.1) and 0.88 (UV5.1). However, the obtained fit statistics suggested the need to improve the model to fit the data more adequately (*TLI* = 0.907, *CFI* = 0.943, *SRMR* = 0.108, *RMSEA* = 0.121).

After inspection of the modification indices, a residual covariance between the items UV5.1 and UV5.2 of the vignette UV5 “Follow-up care” was permitted in Model 2. This addition seems plausible, considering the overlap between the phrasing of the two items (see Additional file 1: Appendix A). The standardized factor loadings for Model 2 ranged between 0.6 (UV4.2) and 0.78 (UV1.2), see Fig. 1. The two factors in Model 2 correlated with *r* = 0.52, *p* ≤ 0.001. The model fit statistics obtained for Model 2 showed improved and adequate model fit: *CFI* = 0.974, *TLI* = 0.954, *RMSEA* = 0.085, *SRMR* = 0.081.

#### Construct validity of the API-Uro

We assessed the construct validity of the disease-specific API-Uro and its two factors (i.e., decision context) with correlation analyses, with the well-established generic API questionnaire [45], as well as with the original case vignettes from the English API (API-OV) [23].

The first factor *treatment decisions* correlated moderately with the generic disease-unspecific API (*r*_study1_ = 0.39 and *r*_study2_ = 0.35), and with the original API vignettes API-OV (*r*_study1_ = 0.38 and *r*_study2_ = 0.33), all correlations where significant (*p* ≤ 0.001).

The second factor *diagnostic decisions* of the API-Uro, also correlated moderately with the generic API (*r*_study1_ = 0.33 and *r*_study2_ = 0.34), but strongly with the generic vignettes API-OV (*r*_study1_ = 0.63 and *r*_study2_ = 0.60). Again, all correlations were significant below a threshold of *p* ≤ 0.001.

### Context specificity and urooncological participation preference

In order to establish the context specificity of the API-Uro, and highlight the relevance of assessing patients’ preference for active participation in the specific decision contexts, we compared the participation preference for *treatment decisions* vs. *diagnostic decisions* in both studies. Patients were categorized based on a taxonomy from the literature [30, 50, 62] into either delegators (participation preference ≤ 40) or participators (participation preference > 40). Table 5 illustrates the differences in participation preference depending on the decision context and the respective diagnoses of the patients.

**Table 5:** Participation Preference (API-Uro) of the Urological Cohort (Study 1) and the Bladder Cancer Cohort (Study 2)

Measure	Participators* Treatment Decisions*		Participators* Diagnostic Decisions*		χ^2^(1)^a^	*p*
	*n*	*%*	*n*	*%*		
*Urological Cohort (Study 1)*	350	77.8	99	22.0	245.1	≤ .001
Non-Oncological	199	77.1	60	23.3	137	≤ .001
Prostate Cancer	96	76.8	24	19.2	70.1	≤ .001
Bladder Cancer	28	80.0	7	20.0	19.2	≤ .001
Other Urooncological Cancer	23	85.2	8	29.6	15	≤ .001
*Bladder Cancer Cohort (Study 2)*	112	57.4	26	13.3	84	≤ .001

Differing numbers to the reported total sample sizes *N*_*1*_ = 469 and *N*_*2*_ = 204 are due to missings values still present after median imputation

^a^McNemar χ^2^ test

In the *Urological Cohort (Study 1),* the proportion of self-identified participators was independent of the primary diagnoses; however, there were substantial differences in the proportion of participators between the specific decision context (i.e., if the decisions were pertaining to a treatment decision or a diagnostic decision). Overall, a majority (76.8%—85.2%) had a desire to participate when it came to urooncological treatment decisions. In contrast, in the context of diagnostic decisions, only about a quarter of patients (22%—29.6%) reported a desire to participate.

The pattern of a substantially (*p* ≤ 0.001) higher proportion of participators for treatment decisions than for diagnostic decisions was replicated in the *Bladder Cancer Cohort (Study 2)*. Again, a majority of 57.4% self-identified as participators for treatment decisions as opposed to only 13.3% for diagnostic decisions.

## Discussion and conclusions

The key to the successful implementation of shared decision-making (SDM) is to accurately identify patients’ desire to participate in decision-making [14, 71]. Especially in cases where the stakes are high (typical in the treatment of urological cancer) and when the best decision heavily depends on the patient’s preferences [16], there is a need for psychometrically validated measures of participation preference.

Our findings demonstrated the importance of assessing patients’ preference for participation in the specific decision context of the current treatment. Overall, most urological and urooncological patients desired active participation in *treatment* but not in *diagnostic decisions*, highlighting the relevance of context-specific assessment of patients’ preferences in the field.

Differences in the participation preference between the two decision contexts might be due to the subjectively perceived difficulty or stakes of the decisions they comprise. The observed higher participation preference for the more difficult and complex *treatment decisions* compared to the lower-stakes *diagnostic decisions* might, at first glance, appear to contradict previous findings of lower participation preference in vignettes with more severe diseases [23, 72, 73]. However, we argue that it is important to disentangle the influences of illness severity and decision difficulty on participation preference.

While there is some evidence for a lower participation preference in patients with more severe illness, more comorbidities, lower physical functioning, or health-related quality of life [32, 73–75], we did not find any relationship between participation preference and illness severity in our *urological cohort (Study 1)*. Table 5 illustrates that oncological patients did not differ from their non-oncological counterparts.

However, we found a substantial effect of the specific decision contexts (*treatment* vs. *diagnostic decisions*) on participation preference. This is in line with a previous study in which participants differed significantly in their participation preference in generic medical decision-making compared to decisions regarding a specific disease [26]. Furthermore, O’Dell and colleagues [37] investigated the influence of different decision contexts for prostate cancer screening decisions (PSA-Tests). They reported small differences between the participation preference for decisions about acceptable risks and benefits (71.3% active participation) compared to the decision about what treatment option is selected (64.4% active participation).

Overall, the new disease and context-specific Autonomy Preference Index – Urooncology (API-Uro) has promising psychometric properties and is a suitable tool to assess patients’ desire to participate in two important urooncological decision contexts, namely, *treatment decisions* and *diagnostic decisions*. The two-factor structure obtained in the heterogeneous sample collected in the *urological cohort (Study 1)* explained a substantial proportion of the variance and was replicated with an independent sample from a multicenter trial (*bladder cancer cohort; Study 2)*. Internal consistency for the factors was acceptable to good. The finding of moderate correlations with the established, generic (i.e., independent of the disease and decision context) API questionnaire indicates that the new context-specific API-Uro measures a related but not identical construct, thus, speaking for its convergent construct validity.

Context-specific measures of participation preference, therefore, provide important information and incremental value over generic ones.

### Strengths and limitations

Our study’s strengths include providing data on the participation preference of previously understudied urological cancer populations (e.g., bladder cancer patients) obtained in a multicenter study. Furthermore, the rigorous psychometric evaluation of the new API-Uro questionnaire and the replication of its factorial validity in an independent multicenter sample support the overall quality of the instrument.

However, the model fit for the replication of the factorial structure (with the CFA) was not perfect. This is somewhat expected and a typical problem in factor analysis methodology due to the fixation of small factor patterns in exploratory factor analysis (omission of loadings < 0.3; [76]). We optimized the fit by permitting a covariance between the residuals of two items with high similarity on the *diagnostic decisions* subscale. Thus reaching acceptable to good model fit on most fit statistics [77].

One general criticism of the measurement of preference prior to the consultation in which high-stake decisions are to be made is that patients may not be capable of fully anticipating the task ahead. If they are currently not affectively aroused (in a “cold” state, as when feeling calm while filling in a questionnaire), it may be difficult to imagine being in an affective aroused state (in a “hot state”, as when feeling anxious during the decision-making). Patients might consequently underestimate the motivational influence of the hot state on their preferences (cold-to-hot empathy gap) [30, 78]. Asking patients for their participation preferences before they are entirely aware of the possible options also forces them to prejudge their preferences [13].

The chosen format of the API-Uro to assess participation preference and presenting options in a vignette format could hold some merit and help counteract this problem. In addition, the temporal proximity of preference assessment directly before the decision situation in the presented studies might attenuate the expected effect of empathy gaps. In future studies, a direct pre-post consultation comparison of context-specific participation preference might help elucidate how much such empathy gaps impact patients’ preferences.

Another limitation is the systematic drop-out of non-German nationals and patients with lower education in our *urological cohort (Study 1)*. This is problematic as it could indicate a language or cultural barrier to enrollment in our study. However, this study’s overall drop-out rate was low (6.6%), and the pattern did not occur in the *bladder cancer cohort (Study 2),* so that we trust the results to be reasonably representative.

## Conclusion

Truly individualized care does not mean that every patient should be pushed to participate in decisions on every aspect of their care. Respecting patient autonomy includes identifying patients who do not wish to participate and to accommodate this preference as well [79]. Thus, for SDM to be successful, it is essential to validly and reliably assess patients’ preference for participation [33, 80]. Indeed, we found substantial differences in patients’ participation preferences depending on the specific decision contexts. Patients’ participation preference was substantially higher for *treatment* than for *diagnostic decisions*. This showcases the incremental value context-specific measures of participation preference.

Since the positive effects of patient involvement in SDM may only be achieved when there is a reasonable match between the preferred extent of participation and the perceived level of involvement [81–83], a precise and specific measurement tool is critical.

The newly developed API-Uro has good psychometric properties and may prove a valuable tool for SDM research and clinical practice. It could enable healthcare providers and researchers to adequately assess urooncological patients’ desire to participate in the two major decision contexts: *treatment* and *diagnostic decisions*. Given the substantial difference in participation preference between these two decision contexts, we recommend calculating separate scores on the two factors rather than on a total sum score only. Integration of the psychometrically validated, disease and context-specific API-Uro in clinical practice could enable the involvement of patients in agreement with their preferences and thus to better meet their needs.

## Supplementary Information

**Additional file 1:** **Appendix A.** English version of the final API-Uro questionnaire. **Figure B.1.** Urological cohort - pattern of missing values in the API-OV. **Figure B.2.** Urological cohort - pattern of missing values in the API-Uro. **Figure B.3.** Bladder cancer cohort - missing values in API-OV. **Figure B.4.** Bladder cancer cohort - pattern of missing values in API-Uro. **Figure D.1.** Scree plot from parallel analysis.

## Data Availability

The data and R code that support the findings of this study will be deposited on MADATA (University of Mannheim, https://madata.bib.uni-mannheim.de/) Research Data Repository (doi: 10.7801/419) and made available by the authors, without undue reservation, to any qualified researcher.

## Abbreviations

API: Autonomy Preference Index
API-dm: Decision-making (subscale of the API)
API-is: Information-seeking (subscale of the API)
API-OV: Autonomy Preference Index – original case vignettes
API-Uro: Autonomy Preference Index – urooncology
CFA: Confirmatory factor analysis
CFI: Comparative fit index
DWLS: Diagonally weighted least squares
EFA: Exploratory factor analysis
RMSEA: Root mean square error of approximation
SDM: Shared decision-making
SRMR: Standardized root mean square residual
TLI: Tucker–Lewis index
UV_i.k_: Urological Vignette, i indicates the vignette, k the item on the respective vignette
