# Supplementary material for: Getting specific: participation preference in urooncological decision-making
Source: BMC Med Inform Decis Mak. 2023 Jul 6;23:114. doi: 10.1186/s12911-023-02201-8 (PMC10324222; doi:10.1186/s12911-023-02201-8)
Supplement: Supplementary file 1 — Additional file 1: Appendix A. English version of the final API-Uro questionnaire. Figure B.1. Urological cohort - pattern of missing values in the API-OV. Figure B.2. Urological cohort - pattern of missing values in the API-Uro. Figure B.3. Bladder cancer cohort - missing values in API-OV. Figure B.4. Bladder cancer cohort - pattern of missing values in API-Uro. Figure D.1. Scree plot from parallel analysis. [file 12911_2023_2201_MOESM1_ESM.docx]

# Appendix A – English version of the final API-Uro questionnaire

The German version of the API-Uro questionnaire is available at the repository of the University of Mannheim and can be obtained from <https://madoc.bib.uni-mannheim.de/59831/> (Büdenbender et al., 2021).

### API-Uro – Urooncological Case Vignettes

##

This questionnaire aims to assess your desire for participation in oncology. To this end, you will be presented with the fictional case of patient P.

Initially, P. has a routine medical check-up during which P is diagnosed with malignant urological **cancer**. Over the course of cancer treatment, different decisions need to be made.

Please try to take P’s perspective at each step of the treatment process and indicate who should make the upcoming decision **in your opinion**. There are no right or wrong answers.

**Important:** When filling out this questionnaire, please try to put yourself in P.'s position as well as possible.

**1. Screening**

During a routine check-up, the general practitioner asks Mr. / Mrs. P. about certain complaints that may be early signs of malignant cancer and performs a physical examination.

| *In your opinion, who should  decide …* | The doctor alone | | Mostly the doctor | Doctor and patient equally | Mostly the patient | The patient alone |
| --- | --- | --- | --- | --- | --- | --- |
| whether other procedures, such as a blood test or an ultrasound examination, are also applied? |  |  | |  |  |  |
| whether there is an additional check-up with a urologist? |  |  | |  |  |  |

**2. Functional Preservation vs. Oncological Safety.**

To treat P. a surgery needs to be performed. The surgery can be **extensive** (radical) in order to have the highest chance of completely removing the tumor. Alternatively, the operation can be performed **function-sparing** (in an attempt to better preserve sexual function or continence, for example). In this case, there is a higher risk for tumor tissue to remain in the body.

| *In your opinion, who should  decide …* | The doctor alone | Mostly the doctor | Doctor and patient equally | Mostly the patient | The patient alone |
| --- | --- | --- | --- | --- | --- |
| whether an “radical” or “function-sparing” operation is carried out? |  |  |  |  |  |

**3. Adjuvant Chemotherapy**

After the surgery, it is possible to administer **additional chemotherapy** to r**educe the risk of recurrence of the disease.** Chemotherapy may be associated with **side effects** such as pronounced fatigue or nausea, hair loss or immune deficiency.

| *In your opinion, who should  decide …* | The doctor alone | Mostly the doctor | Doctor and patient equally | Mostly the patient | The patient alone |
| --- | --- | --- | --- | --- | --- |
| whether chemotherapy is administered? |  |  |  |  |  |
| when to start it? |  |  |  |  |  |

**4. Aftercare**

| *In your opinion, who should  decide …* | The doctor alone | Mostly the doctor | Doctor and patient equally | Mostly the patient | The patient alone |
| --- | --- | --- | --- | --- | --- |
| whether follow-up checks are necessary? |  |  |  |  |  |
| if so, how often should these take place? |  |  |  |  |  |

**How well were you able to take patient P’s perspective?**

Not well at all

Very well

**Literature**

Büdenbender, B., Kriegmair, M. C., Köther, A. K., Grüne, B., Michel, M. S., & Alpers, G. W. (2021). *Autonomy Preference Index - Uroonkologie (API-Uro)*. University of Mannheim. <https://madoc.bib.uni-mannheim.de/59831/>

# Appendix B – Patterns of missing values

**Figure B.1***Urological cohort (Study 1)* - missing values in API-OV





**Figure B.2**

*Urological cohort (Study 1)* - missing values in the initial version of the API-Uro (12-Items)





**Figure B.3**

*Bladder cancer cohort (Study 2)* - missing values in API-OV





**Figure B.4**

*Bladder cancer cohort (Study 2)* - missing values in the initial version of the API-Uro (12-Items)





# Appendix C – Full list of R-packages

| Packagename | Version | Maintainer | Reference |
| --- | --- | --- | --- |
| chisq.posthoc.test | 0.1.2 | Daniel Ebbert <daniel.ebbert@uni-muenster.de> | Ebbert, D. (2019). *chisq.posthoc.test: A Post Hoc Analysis for Pearson’s Chi-Squared Test for Count Data*. <http://chisq-posthoc-test.ebbert.nrw/> |
| colorspace | 2.0.2 | Achim Zeileis <Achim.Zeileis@R-project.org> | Zeileis, A., Hornik, K., & Murrell, P. (2009). Escaping {RGB}land: Selecting Colors for Statistical Graphics. *Computational Statistics \& Data Analysis*, *53*(9), 3259–3270. <https://doi.org/10.1016/j.csda.2008.11.033> |
| conflicted | 1.0.4 | Hadley Wickham <hadley@rstudio.com> | Wickham, H. (2019). *conflicted: An Alternative Conflict Resolution Strategy*. <https://github.com/r-lib/conflicted> |
| effectsize | 0.6.0.1 | Mattan S. Ben-Shachar <matanshm@post.bgu.ac.il> | Ben-Shachar M, Lüdecke D, Makowski D (2020). effectsize: Estimation of Effect Size Indices and Standardized Parameters. Journal of Open Source Software, 5(56), 2815. doi: 10.21105/joss.0281 |
| datscience | 0.2.3 | Björn Büdenbender <b_buedenbender@yahoo.de> | Büdenbender, B. (2022). *datscience: Data Science Utility Functions*. <https://github.com/Buedenbender/datscience/> |
| DescTools | 0.99.43 | Andri Signorell <andri@signorell.net> | Signorell, A. (2021). *DescTools: Tools for Descriptive Statistics*. <https://cran.r-project.org/package=DescTools> |
| dplyr | 1.0.7 | Hadley Wickham <hadley@rstudio.com> | Wickham, H., François, R., Henry, L., & Müller, K. (2021). *dplyr: A Grammar of Data Manipulation*. <https://cran.r-project.org/package=dplyr> |
| forcats | 0.5.1 | Hadley Wickham <hadley@rstudio.com> | Wickham, H. (2021). *forcats: Tools for Working with Categorical Variables (Factors)*. <https://cran.r-project.org/package=forcats> |
| ggplot2 | 3.3.5 | Thomas Lin Pedersen <thomas.pedersen@rstudio.com> | Wickham, H., Chang, W., Henry, L., Pedersen, T. L., Takahashi, K., Wilke, C., Woo, K., Yutani, H., & Dunnington, D. (2021). *ggplot2: Create Elegant Data Visualisations Using the Grammar of Graphics*. <https://cran.r-project.org/package=ggplot2> |
| ggpubr | 0.4.0 | Alboukadel Kassambara <alboukadel.kassambara@gmail.com> | Kassambara, A. (2020). *ggpubr: ggplot2 Based Publication Ready Plots*. <https://rpkgs.datanovia.com/ggpubr/> |
| glue | 1.4.2 | Jim Hester <james.f.hester@gmail.com> | Hester, J. (2020). *glue: Interpreted String Literals*. <https://cran.r-project.org/package=glue> |
| gmodels | 2.18.1 | Gregory R. Warnes <greg@warnes.net> | Warnes, G. R., Bolker, B., Lumley, T., & Johnson, R. C. (2018). *gmodels: Various R Programming Tools for Model Fitting*. <https://cran.r-project.org/package=gmodels> |
| haven | 2.4.3 | Hadley Wickham <hadley@rstudio.com> | Wickham, H., & Miller, E. (2021). *haven: Import and Export SPSS, Stata and SAS Files*. <https://cran.r-project.org/package=haven> |
| Hmisc | 4.5.0 | Frank E Harrell Jr <fh@fharrell.com> | Harrell Jr., F. E. (2021). *Hmisc: Harrell Miscellaneous*. <https://cran.r-project.org/package=Hmisc> |
| labelled | 2.8.0 | Joseph Larmarange <joseph@larmarange.net> | Larmarange, J. (2021). *labelled: Manipulating Labelled Data*. <http://larmarange.github.io/labelled/> |
| lavaan | 0.6.9 | Yves Rosseel <Yves.Rosseel@UGent.be> | Rosseel, Y. (2012). lavaan: An R Package for Structural Equation Modeling. *Journal Of Statistical Software*, *48*(2), 1–36. <https://doi.org/10.18637/jss.v048.i02> |
| lavaanExtra | 0.0.3 | Rémi Thériault <remi.theriault@mail.mcgill.ca> | Thériault, R. (2022). *lavaanExtra: Convenience Functions for Package `lavaan`*. <https://lavaanextra.remi-theriault.com> |
| MVN | 5.9 | Selcuk Korkmaz <selcukorkmaz@gmail.com> | Korkmaz, S., Goksuluk, D., & Zararsiz, G. (2021). *MVN: Multivariate Normality Tests*. <https://cran.r-project.org/package=MVN> |
| pacman | 0.5.1 | Tyler Rinker <tyler.rinker@gmail.com> | Rinker, T., & Kurkiewicz, D. (2019). *pacman: Package Management Tool*. <https://github.com/trinker/pacman> |
| psych | 2.1.9 | William Revelle <revelle@northwestern.edu> | Revelle, W. (2021). *psych: Procedures for Psychological, Psychometric, and Personality Research*. <https://personality-project.org/r/psych/> |
| purrr | 0.3.4 | Lionel Henry <lionel@rstudio.com> | Henry, L., & Wickham, H. (2020). *purrr: Functional Programming Tools*. <https://cran.r-project.org/package=purrr> |
| rcompanion | 2.4.1 | Salvatore Mangiafico <mangiafico@njaes.rutgers.edu> | Mangiafico, S. (2021). *rcompanion: Functions to Support Extension Education Program Evaluation*. <http://rcompanion.org/> |
| readr | 2.0.2 | Jim Hester <jim.hester@rstudio.com> | Wickham, H., & Hester, J. (2021). *readr: Read Rectangular Text Data*. <https://cran.r-project.org/package=readr> |
| rvest | 1.0.1 | Hadley Wickham <hadley@rstudio.com> | Wickham, H. (2021). *rvest: Easily Harvest (Scrape) Web Pages*. <https://cran.r-project.org/package=rvest> |
| semPlot | 1.1.2 | Sacha Epskamp <mail@sachaepskamp.com> | Epskamp, S. (2019). *semPlot: Path Diagrams and Visual Analysis of Various SEM Packages’ Output*. <https://github.com/SachaEpskamp/semPlot> |
| sjlabelled | 1.1.8 | Daniel Lüdecke <d.luedecke@uke.de> | Lüdecke, D. (2021). *sjlabelled: Labelled Data Utility Functions (Version 1.1.8)*. <https://doi.org/10.5281/zenodo.1249215> |
| stringr | 1.4.0 | Hadley Wickham <hadley@rstudio.com> | Wickham, H. (2019). *stringr: Simple, Consistent Wrappers for Common String Operations*. <https://cran.r-project.org/package=stringr> |
| survival | 3.2.11 | Terry M Therneau <therneau.terry@mayo.edu> | Therneau, T. M. (2021). *survival: Survival Analysis*. <https://github.com/therneau/survival> |
| table1 | 1.4.2 | Benjamin Rich <mail@benjaminrich.net> | Rich, B. (2021). *table1: Tables of Descriptive Statistics in HTML*. <https://github.com/benjaminrich/table1> |
| tibble | 3.1.4 | Kirill Müller <krlmlr+r@mailbox.org> | Müller, K., & Wickham, H. (2021). *tibble: Simple Data Frames*. <https://cran.r-project.org/package=tibble> |
| tidyr | 1.1.4 | Hadley Wickham <hadley@rstudio.com> | Wickham, H. (2021). *tidyr: Tidy Messy Data*. <https://cran.r-project.org/package=tidyr> |
| tidyverse | 1.3.1 | Hadley Wickham <hadley@rstudio.com> | Wickham, H. (2021). *tidyverse: Easily Install and Load the Tidyverse*. <https://cran.r-project.org/package=tidyverse> |
| VIM | 6.1.1 | Matthias Templ <matthias.templ@gmail.com> | Templ, M., Kowarik, A., Alfons, A., de Cillia, G., & Rannetbauer, W. (2021). *VIM: Visualization and Imputation of Missing Values*. <https://github.com/statistikat/VIM> |

# Appendix D – Parallel analysis and screeplot

**Figure D.1**

*Scree plot from parallel analysis*





*Note.* Observed Eigenvalues plotted against simulated data. Parallel analysis identified five factors to be extracted (dashed line). Scree test (break or point of maximum inflection) identified two factors.
